# White blood cell counts have an impact on septic patient outcome followed by polymyxin-b immobilized fiber with direct hemoperfusion

**DOI:** 10.1186/cc14208

**Published:** 2015-03-16

**Authors:** H Tanaka, T Ikeda, S Ono, S Suda, T Ueno

**Affiliations:** 1 Tokyo Medical University, Hachioji Medical Center, Hachioji, Japan

## Introduction

The mortality rate of severe sepsis and septic shock is varied and high (25 to 70%). In our institute, the indication for polymyxin-B immobilized fiber with direct hemoperfusion (PMX-DHP) has been that circulatory failure (systolic blood pressure <90 mmHg or required catecholamines and high lactacidemia) continued despite following early goal-directed therapy by the Surviving Sepsis Campaign guidelines 2012.

## Methods

This study included 80 patients with severe sepsis or septic shock due to abdominal infection retrospectively. These subjects were divided into two groups: those with WBC counts <4,000 (L-group: 64 patients) and those with WBC counts >12,000 (H-group: 16 patients). Mean arterial pressure, WBC counts, platelet counts, interleukin-6 (IL-6), and plasminogen activator inhibitor-1 (PAI-1) were measured immediately before the initiation and after the completion of PMXDHP. Statistical analysis was performed using the chi-squared test for background factors, with Wilcoxon's rank-sum test for comparison within a group, and Mann-Whitney's *U *test for comparison between groups. The significance level was set at *P *<0.05.

## Results

The mortality rate of 28 days in the L-group was 32.8%, and was 18.8% in the H-group. Mean arterial pressure increased significantly (*P *<0.01) in the H-group compared with the L-group. WBC counts in the L-group increased and in the H-group decreased (*P *<0.01) during PMXDHP treatment. Platelet counts in both groups decreased significantly (*P *<0.01).There was no significant difference between before and after PMX-DHP in IL-6 levels. On the other hand, IL-1ra decreased significantly before and after PMX-DHP. Also, IL-6 and IL-1ra in the L-group were significantly higher than those in the H-group at the start of PMXDHP. PCT values in the L-group were increased compared with the H-group at the start of PMX-DHP (*P *<0.01). PCT in the L-group increased significantly (*P *<0.01), but no significant changes in the H-group. PAI1 showed no significant changes before and after PMX-DHP and no changes in both groups at the start of PMX-DHP.

## Conclusion

The mortality rate of the L-group tended to be higher than that of the H-group. Inflammatory and anti-inflammatory cytokines in the L-group were higher than those of the H-group. These results indicate that leukopenia (WBC <4,000) in severe sepsis patients leads to more severe outcome and hypercytokinemia than leukocytosis (WBC >12,000) in severe sepsis patients.

